# Neural stem cell therapy for subacute and chronic ischemic stroke

**DOI:** 10.1186/s13287-018-0913-2

**Published:** 2018-06-13

**Authors:** Austin C. Boese, Quan-Son Eric Le, Dylan Pham, Milton H. Hamblin, Jean-Pyo Lee

**Affiliations:** 1 Department of Physiology, Tulane University School of Medicine, New Orleans, LA 70112 USA; 2 Department of Pharmacology, Tulane University School of Medicine, New Orleans, LA 70112 USA; 3 Center for Stem Cell Research and Regenerative Medicine, Tulane University School of Medicine, New Orleans, LA 70112 USA

**Keywords:** Blood-brain barrier, Neural stem cells, Stroke, Tissue plasminogen activator, Transplantation

## Abstract

Neural stem cells (NSCs) play vital roles in brain homeostasis and exhibit a broad repertoire of potentially therapeutic actions following neurovascular injury. One such injury is stroke, a worldwide leading cause of death and disability. Clinically, extensive injury from ischemic stroke results from ischemia-reperfusion (IR), which is accompanied by inflammation, blood-brain barrier (BBB) damage, neural cell death, and extensive tissue loss. Tissue plasminogen activator (tPA) is still the only US Food and Drug Administration–approved clot-lysing agent. Whereas the thrombolytic role of tPA within the vasculature is beneficial, the effects of tPA (in a non-thrombolytic role) within the brain parenchyma have been reported as harmful. Thus, new therapies are needed to reduce the deleterious side effects of tPA and quickly facilitate vascular repair following stroke. The Stroke Treatment Academic Industry Roundtable (STAIR) recommends that stroke therapies “focus on drugs/devices/treatments with multiple mechanisms of action and that target multiple pathways”. Thus, based on multifactorial ischemic cascades in various stroke stages, effective stroke therapies need to focus on targeting and ameliorating early IR injury as well as facilitating angiogenesis, neurogenesis, and neurorestorative mechanisms following stroke. This review will discuss the preclinical perspectives of NSC transplantation as a promising treatment for neurovascular injury and will emphasize both the subacute and chronic phase of ischemic stroke.

## Background

According to the Centers for Disease Control and Prevention, stroke is a leading cause of death in the United States [1], and ischemic stroke incidents comprise nearly 90% of all strokes [1]. Aging is a major risk factor, and overall, two thirds of strokes occur in patients over 65 years old [2]. Currently, we live in an aging nation with more than 45 million Americans who are at least 65 years old. By 2060, these numbers will likely have doubled.

Despite the high incidence of stroke and cerebrovascular disease, effective treatments and therapies are limited. Currently, therapy for ischemic stroke is limited to fast recanalization (thrombectomy) and tissue plasminogen activator (tPA), a compound that breaks apart thrombi in cerebral arteries to restore blood flow [3]. Major limitations to tPA treatment include a narrow effective therapeutic window of 4.5 h after initial stroke [4] and a high potential for hemorrhagic transformation [5]. Therefore, new treatment strategies are needed to increase the narrow therapeutic window of tPA, minimize detrimental side effects, and improve patient outcome following stroke. Of note, the pathophysiology of ischemic stroke is incredibly complex, and neural stem cells (NSCs) show pleiotropic effects that are potentially therapeutic for both the early (subacute) phase and chronic phase of stroke. These include functional neural replacement in multiple central nervous system (CNS) regions [6] or bystander effects that include delivery of NSC-synthesized therapeutic gene products, which could both directly protect the endangered host cells and inhibit toxic components of the microenvironment [7–11]. For example, a growing body of preclinical research suggests that NSC transplantation is an effective therapy for ischemic stroke through multiple mechanisms, such as preservation of the blood-brain barrier (BBB), alleviation of neuroinflammation, enhanced neurogenesis and angiogenesis, and ultimately functional neurological recovery [12, 13]. Furthermore, animal studies on NSC transplantation report different therapeutic effects depending on the stage of disease and route of administration. Therefore, this review is to highlight the therapeutic effects of NSC transplantation in various chronological stages of ischemic stroke. Stem cell delivery in subacute stroke may benefit more patients by ameliorating early-phase stroke injury, thus reducing later complications of secondary damage. This review will provide (1) an overview of NSC biology, including sources of NSCs and tracking of NSCs and their distribution, and (2) the complex pathophysiological cascades following ischemic stroke and impact of NSC delivery at these distinct stages of stroke.

## Biology of neural stem cells

### Endogenous neural stem cells

NSCs are the least committed cells of the nervous system and have the functional properties of self-renewal and multipotency to generate all three fundamental neuroectodermal lineages. NSCs generate neurons, astrocytes, and oligodendrocytes in a regional and developmental stage-appropriate manner throughout life. In mammals, neurogenesis occurs throughout life in localized brain regions called “niches” where NSCs are present [14]. These regions include the subgranular zone of the hippocampal dentate gyrus [15], the subventricular zone (SVZ) of the lateral ventricles [16–18], and the external germinal layer (EGL) of the cerebellum [19]. The EGL disappears once cerebellar development is completed, by 3 weeks postnatally in rodents [20] and by 2 years of age in humans [21]. In rodents, there is substantial evidence that neural progenitors in the SVZ proliferate throughout life, differentiate, and migrate through a track called the rostral migratory system to become new functional neurons in the olfactory bulb (OB) [14, 22]. However, recent studies suggest that humans and laboratory rodents differ in regard to the magnitude and duration of niche-specific adult neurogenesis. There is ample evidence that neurogenesis persists in the hippocampus [23, 24] and striatum [25] throughout aging in humans. Although the presence of NSCs in the adult human SVZ has been confirmed [26], postnatal migration of neuroblasts from this neurogenic niche to the OB may not occur in humans [27, 28]. Animal studies report that neurogenesis is very low by middle age [29], and human data support that hippocampal neurogenesis diminishes sharply over time [30].

In healthy adult mammals, neurogenesis occurs at a very limited rate [14, 31]. However, injury to the CNS stimulates neurogenesis. Pathological insults such as ischemia lead to increased proliferation, migration, and differentiation of NSCs and neural progenitors in the brain [32, 33]. Studies on adult humans have reported increased neural progenitor cells in brain parenchyma proximal to the ventricle wall and cells expressing Ki67, a marker of proliferation, in the ipsilateral SVZ following ischemic insult [34, 35]. Unfortunately, endogenous neurogenesis does not supply enough cells to repair neurological damage from major events like stroke [33]. Therefore, transplantation of cultured NSCs to aid endogenous neural progenitors could be an effective therapy to repair CNS damage from stroke.

### Deriving neural stem cells in vitro

**Fig. 1:**
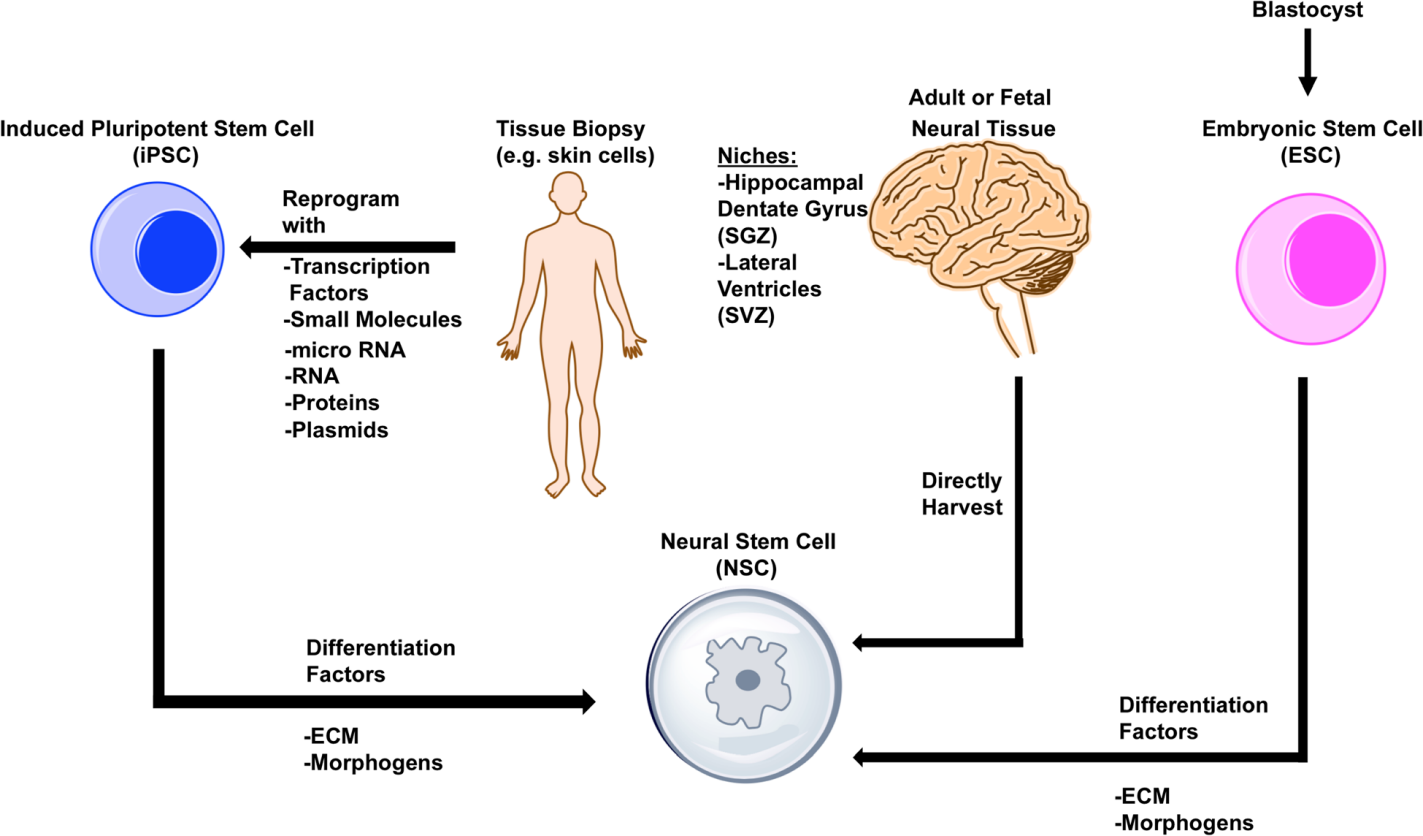
Schematic diagram of NSC generation via multiple methods. NSCs can be extracted directly from neurogenic niches, including the subgranular zone (SGZ) of the hippocampal dentate gyrus and subventricular zone (SVZ) of the lateral ventricles, from adult or fetal neural tissue and expanded in vitro. NSCs can be reprogrammed from patient-derived induced pluripotent stem cells by using combinations of specific transcription factors, small molecules, microRNAs, plasmids, and other morphogens. In addition, NSCs can be generated from blastocyst-derived embryonic stem cells by using specific combinations of differentiation factors, such as morphogens and extracellular matrix (ECM) proteins. *Abbreviation*: *NSC* neural stem cell

In order for NSC transplantation to be a viable therapy for stroke patients in the clinic, these cells must be able to be successfully cultured and expanded ex vivo. In fact, there are many ways to derive NSCs in vitro (Fig. 1). Since neurogenesis occurs throughout life in mammals, NSCs can be extracted directly from neural tissue, such as the neuroectoderm in developing fetuses or the subventricular or subgerminal zone in adults [36]. These NSCs can be propagated in vitro by using serum-free media with basic fibroblast growth factor (bFGF) [37–39] and epidermal growth factor (EGF) supplementation [36, 37, 40].

Isolation and propagation of NSCs can also be accomplished through other in vitro methods. For instance, NSCs can be derived from embryonic stem cells (ESCs) [41–43]. These cells originate from the inner cell mass of blastocysts and can give rise to progeny that can differentiate into any somatic cell type. One limitation of this approach is that ESCs require a great deal of manipulation to fully commit their fate toward differentiating into NSCs [41, 43]. Neuroinduction of ESCs can be accomplished by blocking transforming growth factor-beta/bone morphogenic protein (TGF-β/BMP) signaling pathways while promoting expansion with bFGF or EGF [44]. In order to minimize tumorigenic risk of undifferentiated cells, in vitro culturing time for ESC-derived NSCs is usually lengthened [45, 46].

NSCs can be similarly derived from human induced pluripotent stem cells (iPSCs) [44, 47]. Many types of somatic cells have been reprogrammed to generate iPSCs. These include fibroblasts, which can easily be obtained from human biopsies. Of note, the same method of dual-inhibiting SMAD signaling for ESCs can be used to transform iPSCs into NSCs [44]. Therefore, it is generally assumed that the same protocols for ESCs can be used to differentiate iPSCs into NSCs. However, generating iPSCs requires the extra, lengthy step of reprogramming already-differentiated somatic cells back to an undifferentiated state [48]. In vitro studies using microarray analysis have confirmed that iPSC-derived NSCs have very similar but not identical genetic expression compared with ESC-derived NSCs [49, 50]. Some advantages to using iPSCs are that they present fewer ethical concerns and fewer immune issues since they can be extracted and reprogrammed from a patient’s own tissue [47]. Therefore, iPSC-NSCs may have better potential as a treatment for CNS injury. NSCs derived in this manner have been tested in animal models of neurological disease and have proven to be therapeutic.

Also, methods have been developed to reprogram already-differentiated somatic cells into NSCs in a single step through the use of defined growth factors. For instance, experiments have successfully shown that adult fibroblasts can be successfully transformed into NSCs and neural progenitor cells by using the reprograming factors Oct4, Sox2, Klf4, and c-Myc [51]. The resulting induced NSCs exhibit morphology and molecular features similar to those of NSCs generated from other in vitro methods [52]. Similar results have been achieved with different combinations of transcription factors as well [53]. This method of generating NSCs in vitro is advantageous because the lengthy intermediate step of reprogramming somatic cells to iPSCs is skipped altogether. Therefore, direct differentiation of somatic cells to NSCs can save time without sacrificing the therapeutic quality of the manipulated cells. This technique also greatly reduces the risk of teratoma formation through the absence of undifferentiated iPSCs remaining in cell grafts following transplantation [52]. Additionally, direct differentiation of a patient’s own cells to NSCs can eliminate the risk of immune rejection and serve as a source of stem cells that can become neurons since other adult human stem cell sources have shown limited capabilities of fully differentiating into neural cell types [54]. For these reasons, the recent advancements in direct differentiation of stable and expandable NSCs from adult somatic cells are promising for therapeutic applications [55].

### Labeling and tracking exogenous NSCs in vivo

Much of the preclinical research regarding NSC transplantation as a potential therapy for ischemic stroke relies on accurate identification and tracking of engrafted cells to assess their activity in vivo. There are a variety of different methods that investigators can use for labeling NSCs and tracking them after transplantation. One common method for pre-labeling NSCs involves the use of the compound bromodeoxyuridine (BrdU). This molecule incorporates into cellular DNA during the S phase of NSCs in vitro. Stem cells pre-labeled with BrdU can be identified via immunohistochemistry in fixed tissue by using anti-BrdU fluorescent-tagged antibodies or staining methods that use color-changing substrates [36]. Pre-labeling with BrdU is a useful technique for determining the number of cellular divisions that NSCs and their progenitors have undergone in vivo after transplantation [36]. However, this method of stem cell tracking is limited to short-term studies since the BrdU marker becomes increasingly diluted as transplanted stem cells continue to divide [36]. To monitor the migration and survival of engrafted NSCs in vivo non-invasively, human NSCs (hNSCs) can be labeled with super-paramagnetic iron oxide particles for magnetic resonance imaging (SPIO-MRI) [56].

Other methods for labeling and tracking stem cells in vivo include viral transduction of cells to express markers such as GFP and lacZ before transplantation. However, this method of introducing new genes through viral vectors raises concerns about changing the properties of stem cells and the risk of transgene inactivation [36]. When hNSCs are transplanted into rodent (e.g., species mismatch), hNSCs can be verified by human-specific immunomarkers such as human nuclear antigen or human mitochondria. Stem cells can also be identified by sex-mismatch if male stem cells containing Y chromosomes are transplanted into a female host or vice versa. This method can be especially useful if the graft cells are from the same species as the transplant recipient [36].

### Stem cell migration in vivo

Despite the use of stereotactic transplantation equipment in some preclinical studies, stem cells must be able to navigate through the complex microenvironment of the stroke-damaged CNS in order to reach infarct tissue and maximize their therapeutic effects. In mammalian brains, NSCs demonstrate the ability to naturally migrate to areas of injury and neurodegeneration. NSCs from both humans and animals contain various chemokine receptors that facilitate their migration to areas of upregulated pro-inflammatory chemokine expression in the CNS [57–59]. Previous studies have demonstrated that chemokine stromal cell–derived factor-1 alpha (SDF-1α/CXCL12) interacts with NSC CXCR4 receptors as a key step in mediating migration to injury sites [60–62]. The significance of SDF-1α involvement in cell migration is also reflected in studies that have investigated stem cell homing in cardiovascular and renal disease [63–65]. In addition, in vitro analyses using the Transwell chemotaxis assay have shown that NSCs migrate to areas of higher SDF-1α concentrations [66]. SDF-1α levels increase significantly in the stroke-damaged region of the brain, and stem cells show increased migration to these areas of high SDF-1α concentration [67]. Since the SDF-1α/CRCX4 ligand-receptor interaction is heavily involved in stem cell migration in many forms of human disease and injury, manipulating this signaling pathway could also prove to be beneficial in maximizing the amount of NSCs that reach the infarct area of the brain following stroke.

Although the optimal route of NSC delivery remains unresolved, stem cells can be transplanted intracranially into the damaged or penumbral parenchyma [36, 68–70]. Also, NSCs can migrate from intravascular space to the injured brain following intravascular delivery via tail vein [71] or intra-arterial [72] injections. Intravenously administered stem cells can be trapped in filtering organs such as the liver, spleen, and lungs [73–76]. However, intra-arterial administration of stem cells may provide a more direct route to the lesion with better survival and engraftment of donor cells [77, 78].

## Ischemic stroke pathophysiology

The pathophysiology of ischemic stroke is complex and still not completely understood. In addition to the initial ischemic insult, the CNS accumulates additional damage from reperfusion, BBB disruption, and inflammation as time from stroke onset progresses. In order to understand the therapeutic effects of NSC transplantation at the subacute or chronic stroke phase, it is crucial to first understand the pathophysiological cascades that occur with ischemic stroke.

### Blood-brain barrier and reperfusion injury

Integrity of the BBB is very important as it forms a physical barrier created by endothelial tight junction proteins that restrict molecular trafficking through a transcellular route [79, 80]. The BBB is formed from complex interactions between neurons, glial cells, vascular cells, and the extracellular matrix, which are collectively known as the neurovascular unit (NVU). This complex structure maintains brain homeostasis and proper neuronal signaling by regulating the transport of substances into and out of the CNS [81–83]. The BBB also regulates the entry of leukocytes into the CNS for routine immune surveillance and response to infections [84] or after tissue damage when debris needs to be cleared [85].

Ischemic stroke compromises the integrity of the BBB. Ischemia causes metabolic distress due to the deprivation of oxygen and glucose. Energy failure from the depletion of adenosine triphosphate (ATP) stores results in subsequent lactic acidosis, ion transport dysregulation, and the accumulation of extracellular glutamate [86]. These metabolic consequences contribute greatly to endothelial swelling and disruption of the BBB following ischemic insult [87].

Restoration of blood flow is critical to limit damage from ischemic stroke. However, reperfusion of the infarct tissue with oxygenated blood challenges the BBB with oxidative stress and contributes to disruption of the BBB through oxidative damage to cellular molecules, upregulation of inflammatory mediators and matrix metalloproteinases (MMPs), and modulation of tight junction proteins [88–90]. Reperfusion causes a biphasic opening of the BBB, which can cause further damage to the CNS. However, the exact mechanisms underlying increased BBB permeability in the early ischemia-reperfusion (IR) stages are not well understood. The initial opening of the BBB is reversible and occurs within several hours after reperfusion [91], but the second opening is irreversible and occurs 24–72 h after reperfusion [92]. The initial opening is associated with disruption of tight junction proteins from oxidative stress induced after reperfusion [93, 94]. MMPs are able to disrupt tight junction proteins and this makes the BBB leaky and facilitates transport of toxic substances into the ischemic tissue [95]. Later consequences of damage to tight junctions in the BBB include upregulation of pro-inflammatory cytokines and infiltration of peripheral immune cells into the CNS.

In general, the major stages of BBB disruption from reperfusion following ischemic stroke include endothelial swelling, followed by disruption of tight junctions between vascular cells, and eventually complete vascular disruption [96]. Given that the BBB is functionally important for protection against neurotoxic agents and inflammation, preservation of BBB integrity is an attractive therapeutic strategy for ischemic stroke.

### Inflammatory and immune responses

Inflammation is a critical component of the pathophysiology of stroke [97, 98]. Increased permeability of the BBB from reperfusion injury allows peripheral innate and adaptive immune cells, including neutrophils, macrophages, and T cells, to infiltrate the CNS [99]. Although inflammation and immune cell activity both play important roles in wound healing, tissue remodeling, and recovery, a large body of evidence demonstrates that the inflammatory cascade following ischemic stroke can also exacerbate damage to the CNS [100].

Ischemic injury to the CNS triggers the release of inflammatory mediators from dying cells and stimulates an innate immune response [101]. Loss of contact with live neurons and the release of danger-associated molecular pattern molecules following stroke activate resident microglial cells [102–104], which regularly survey the brain for damage. These native, innate immune cells of the CNS exist in two functionally different phenotypic states. Loss of cell-cell contact with healthy neurons [102–104] and receptor activation in response to extracellular glutamate accumulation [105] during ischemic stroke polarize microglia toward the pro-inflammatory M1 phenotype, which secretes factors such as tumor necrosis factor-alpha (TNF-α), interleukin-1 beta (IL-1β), and reactive oxygen species [106]. Alternatively, microglia can polarize toward the M2 phenotype. M2 microglia are beneficial for resolving post-stroke inflammation by secreting anti-inflammatory cytokines such as IL-10 and TGF-β in addition to neurotrophic factors [106, 107].

Less than 24 h after ischemic stroke and subsequent reperfusion injury, peripheral innate immune cells begin to adhere to the damaged endothelium of the BBB and infiltrate the CNS [108]. Macrophages are a major initial infiltrate following stroke [109], and similar to microglia, these immune cells can exhibit functionally different phenotypes [110]. Macrophages with the M1 phenotype worsen damage to the CNS after stroke by secreting pro-inflammatory molecules such as TNF-α, IL-8, and IL-12 [98, 111]. In contrast, macrophages of the M2 phenotype are inflammation-resolving and secrete anti-inflammatory cytokines such as TFG-β and IL-10 [98, 112]. M2 macrophages also participate in the removal of ischemic debris and this phenotype is generally thought to be beneficial for stroke outcome [111]. Infiltrating M1 macrophages recruit neutrophils into the CNS via secretion of IL-8, which contribute to further inflammation and tissue damage by releasing nitric oxide, MMPs, and cathepsins [98]. Infiltration and secretion of factors by peripheral macrophages constitute the acute phase of the inflammatory cascade following ischemic stroke.

The adaptive immune response is also a key component of ischemic stroke pathophysiology. After the infiltration of monocyte-derived macrophages, peripheral CD4^+^ and CD8^+^ T lymphocytes begin to enter the CNS through the disrupted BBB [113, 114]. The accumulation of antigen-presenting cells that express major histocompatibility class II (MHC II) molecule and co-stimulatory molecule CD80 coincides with the height of lymphocyte infiltration in ischemic tissue [115, 116]. Depletion of lymphocytes in mice suggests that T cells, but not B cells, are responsible for further CNS damage in the acute stroke phase [117, 118]. For instance, CNS-specific type 1 T helper (Th1) cells secrete interferon gamma (IFNγ) following ischemic stroke to recruit immune cells into the CNS [119] and activate cytotoxic CD8^+^ T cells, which exacerbate CNS damage by inducing apoptosis via caspase activation and Fas ligand signaling [120]. In addition, γ δ T cells contribute to CNS injury following ischemia through the secretion of the pro-inflammatory cytokine IL-17 [121], while regulatory T cells (T_reg_) are protective through their secretion of IL-10 and TGF-β [122, 123].

Neutrophils are another type of immune infiltrate found in the ischemic hemisphere following reperfusion [124]. However, the arrival of these cells in brain parenchyma is preceded by previously discussed microglia, macrophages, and lymphocytes [115]. The presence of neutrophils relies upon intercellular adhesion molecule 1 (ICAM-1) expression [125] and is associated with increased secretion of proteolytic enzymes and worsened post-ischemic CNS injury in rodent models of stroke [98, 126]. In addition, neutrophil accumulation correlates with poor neurological outcome and severity of brain damage in human stroke patients [127]. Although there have been some reports that blocking neutrophil recruitment in hyperlipidemic mice reduces stroke damage [126], other preclinical studies report no significant effect of neutrophil depletion on ischemic stroke outcome [128].

Neuroinflammation after stroke is self-limited and eventually is resolved when pro-inflammatory mediators become further dampened by secretion of anti-inflammatory molecules, resolvins, and protectins [98]. Although much research has indicated that the inflammatory cascade further contributes to CNS damage, post-stroke neuroinflammation also plays important roles in structural and functional reorganization of the brain after ischemic injury [129]. Clearance of dead cells is completed by an inflammatory response comprised of activated resident microglia and infiltrated peripheral macrophage that gain access to the brain through the choroid plexus-cerebrospinal fluid route [98, 130, 131]. These innate immune cells are attracted to chemokines and purines released from damaged and dying brain cells [132, 133]. Phagocytosis of debris and dying cells promotes upregulation of anti-inflammatory mediators such as TGF-β and IL-10 in activated macrophages and monocytes [134], which help to dampen Th1 and Th2 responses, promote T_reg_ development, and ultimately resolve post-stroke neuroinflammation [135]. Indeed, depletion of monocyte-derived macrophages during the acute post-stroke inflammatory response actually impairs long-term functional recovery in mice [129]. Therefore, immunomodulation to dampen inflammation associated with further CNS damage while preserving immune cell activity responsible for debris clearance and functional remodeling may serve as an excellent strategy for stem cell stroke therapy.

### Tissue loss and behavioral dysfunction

Stroke ultimately leads to extreme tissue death within the CNS. Ischemia results in the depletion of cellular energy stores and this in turn leads to failure of neurons to maintain ionic balance or reuptake of neurotransmitters [136]. In particular, metabolic catastrophe from stroke results in the extracellular accumulation of glutamate, a main excitatory neurotransmitter. Excess extracellular glutamate binds to and activates NMDA and AMPA receptors, thereby promoting a high influx of calcium. Calcium overload in neurons activates proteases and lipases, which degrade cellular organelles and proteins and contribute to cell death [137]. For instance, calcium overload disrupts mitochondria, which house apoptotic proteins. This results in mitochondrial release of cytochrome c and subsequent activation of executioner caspases and downstream cell death pathways in neurons [138, 139].

Edema also contributes to cell death and tissue loss following ischemic stroke. Overactivation of glutamate receptors on neurons promotes the inappropriate influx of sodium and water, thereby causing pathogenic cell swelling [137]. In addition, a compromised integrity of the BBB allows entry of foreign proteins, fluid, and immune cells into the extracellular space within the CNS, which contributes to vasoactive edema and amplifies tissue damage [140]. Vasoactive edema is eventually resolved from post-stroke angiogenesis, but the generation of new blood vessels is too slow to prevent acute edema-associated CNS damage following stroke.

In association with cell death and tissue loss, neural circuitry is also disrupted following stroke. As mentioned above, ischemic insult results in excitotoxicity and sustained depression of inhibitory neurotransmission, primarily through the accumulation of extracellular glutamate [137, 141] and reduction in GABA_A_ receptor expression [142]. This further contributes to behavioral dysfunction and disability after stroke.

## Endogenous repair mechanisms

### Angiogenesis

Although the brain has a limited repair capacity [143], spontaneous stroke recovery occurs [144, 145]. Angiogenesis, the generation of new blood vessels from pre-existing blood vessels, takes place after brain ischemia and is hypothesized to contribute to CNS plasticity and functional recovery [146–149]. Evidence demonstrates that endothelial cell proliferation begins in as little as 12 h following stroke and can persist for several weeks because of the upregulation of growth factors and angiogenic genes [150–152]. Specifically, angiogenesis is stimulated in the ischemic penumbra, which is comprised of tissue proximal to the infarct core and is where endogenous recovery mechanisms take place after ischemic stroke [146]. Higher densities of vessels in the ischemic penumbra are associated with prolonged survival following ischemic stroke [146, 152, 153], and further damage to the penumbra after stroke can have catastrophic consequences for recovery [154].

Angiogenesis has been associated with improved neurological function following stroke. For instance, growth factors that stimulate angiogenesis may also directly promote the survival of cells in the ischemic penumbra [155]. In addition, angiogenesis following ischemic stroke facilitates both increased blood supply and removal of necrotic tissue while establishing a vascular niche for NSC proliferation and migration [156, 157]. However, angiogenic potential can be limited by multiple factors, including aging [158]. Given that ischemic stroke usually occurs later in life, further research on stem cell therapies to improve vascular remodeling after stroke is greatly needed.

### Endogenous neurogenesis and astrogenesis

Ischemic stroke increases endogenous neurogenesis [33], which includes the proliferation and differentiation of NSCs [32, 33, 144]. This process is important in the context of stroke because NSCs can replace damaged neuroblasts in response to stroke injury [159]. Angiogenesis and neurogenesis are coupled in the NVU in that endothelial cells participating in angiogenesis provide growth factors that regulate NSC self-renewal and neurogenesis [160]. The size and density of microvessels change after stroke [161], and neuroblasts from the SVZ migrate close to areas of vascular remodeling [162]. Additionally, NSCs improve angiogenesis via trophic support such as paracrine stimulation with vascular endothelial growth factor (VEGF) and can influence capillary blood flow in the CNS [163, 164]. This highlights a reciprocal relationship between neurogenesis and vascular remodeling after stroke.

Unfortunately, neurogenesis from endogenous NSCs does not supply enough cells to repair neurological damage and newly formed cells may not fully integrate into the neuronal network in ischemic stroke brains [33, 165, 166]. Therefore, transplantation of exogenous NSCs to aid endogenous neural progenitors may be an effective therapy to ameliorate CNS damage from stroke.

Astrocytes also play an important role in angiogenesis and neurogenesis by releasing neurotrophins and vascular growth factors [167, 168]. Astrocyte positioning can also mediate neurotransmission and maintain neuronal activity coupling with cerebral blood vessel activity [169]. In response to acute ischemic injury, astrocytes have neuroprotective effects, such as the formation of glial scars in the ischemic penumbra, uptake of excess glutamate, restriction of the spread of neurotoxic molecules, and increase of revascularization and stabilization of blood vessels in the CNS [170–172]. In response to ischemic injury, there is an increase in production of high thrombospondin-4–expressing (Thbs4hi) astrocytes from NSCs within the SVZ, which is mediated by direct Notch1 signaling and downstream Nfia transcription factor activity [173, 174]. These Thbs4hi astrocytes migrate to the injured cortex following ischemic injury in mice and help to mitigate microvascular hemorrhage into the brain parenchyma [173]. Moreover, Thbs4^−/−^ mice exhibit dysfunctional SVZ astrogenesis and abnormal glial scar formation in addition to extensive, unresolved hemorrhaging in cortical regions following photothrombotic/ischemic injury [173]. Therefore, astrocytes generated from NSCs in the SVZ appear to play an important role in the resolution of BBB disruption in the early stages following stroke. However, in the chronic phase of stroke, the actions of astrocytes can be deleterious because they reduce neurological recovery through secretion of growth inhibitory molecules and glial scar formation, which result in poor connectivity of surviving neural pathways [175]. Thus, targeting early-phase stroke injury with stem cells to enhance endogenous neurogenesis and astrogenesis may reduce later complications of secondary stroke damage.

## Neural stem cell transplantation for ischemic stroke

The environment within the CNS changes dramatically with time following initial ischemic injury. Owing to the complex pathophysiological cascades in ischemic stroke, the timing of NSC transplantation greatly dictates therapeutic mechanisms. Overall, exogenous NSCs may improve neurological outcome following stroke through the replacement of dead or damaged cells or through bystander effects in which NSCs secrete neurotrophic and anti-inflammatory factors to protect brain cells and promote repair.

Replacement of functional neurons and other brain cells is an obvious therapeutic goal of exogenous stem cell transplantation in the stroke-damaged brain. Indeed, grafted NSCs are able to eventually make synaptic connections with host neurons and display electrophysiological properties of mature neurons [176]. However, preclinical research now indicates that bystander effects of exogenous NSCs may be equally or more effective at improving neurological outcome following ischemic insult. It is well established that NSCs provide therapeutic gene products to modify the extracellular microenvironment and promote neuronal circuit plasticity [69, 177, 178]. For example, brain-derived neurotrophic factor (BDNF) is secreted by NSCs. BDNF is a major neurotrophin and promotes neuroprotection, angiogenesis, neurogenesis, and functional recovery after stroke [179–182]. Also, molecular neurotrophic factors (NTFs) help maintain healthy neurons and play important roles in extracellular matrix remodeling, cell generation, and proliferation. NTFs also protect sensitive neural tissue from damage [183]. An important neurotrophic factor that helps promote angiogenesis and also protects and repairs neural tissue is VEGF [155, 184]. VEGF acts as a potent mitogen and survival factor for endothelial cells [185, 186] and has a neuroprotective effect against ischemic injury [155, 187, 188]. When VEGF is inhibited, NSC-mediated protection is significantly decreased [189]. For instance, recent work has demonstrated that NSC-secreted VEGF upregulates glutamate transporter 1 (GLT-1) through PI3-K/Akt pathway activation in astrocytes to facilitate removal of peri-ischemic extracellular glutamate and thereby improve structural and functional plasticity following ischemic insult [190]. Ciliary neurotrophic factor, glial cell line–derived neurotrophic factor, and neural growth factor are other neurotrophic factors that play important roles in maintenance and protection of neural tissue [191–196]. Overall, the therapeutic mechanisms of exogenous NSCs may shift more toward functional cell replacement or bystander effects (or both) depending on whether they are transplanted during the early or chronic stroke phase.

### Transplantation of NSCs at early stroke phase

As mentioned above, reperfusion injury, BBB disruption, and infiltration of immune cells cause further damage to the CNS in the early phase of stroke [88–90, 100]. Most commonly, pharmacological treatments have been employed to reduce acute/subacute stroke damage. For example, minocycline has shown potential to serve as a neuroprotective pharmacological agent for clinical treatment of stroke [197, 198] because of its anti-inflammatory and anti-apoptotic effects and ability to reduce BBB damage [199–203]. However, in some clinical scenarios where ischemic stroke is still in the acute/subacute phase, NSC transplantation may be used as a multifaceted neuroprotective strategy to limit the severity of damage and cell loss from ischemic insult. For instance, studies using the middle cerebral artery occlusion (MCAO) mouse model of ischemic stroke have demonstrated that NSC-engrafted brains 48 h post-stroke (24 h post-transplantation) show decreased inflammation, decreased BBB damage, and consequent improvement of long-term stroke outcome [69, 70, 177, 204–207]. Below are multiple actions of NSCs transplanted in the subacute phase of stroke.

#### BBB support

Early transplantation of NSCs helps to protect against damage to the BBB after ischemic reperfusion. Transplantation of hNSCs into the hippocampus of mice 24 h after MCAO resulted in extensive migration of NSCs to the lesions, reduction in infarct volume, and decreased damage to the BBB compared with untreated MCAO controls [69]. This rapid response suggests that the therapeutic mechanisms of NSCs were anti-inflammatory. In support of this hypothesis, hNSC-transplanted mice showed decreased microglia activation as well as decreased expression of inflammatory factors such as IL-6, IL-1β, and macrophage inflammatory protein-1α [69]. Similarly, transplantation of human iPSC (hiPSC)-NSCs into a stroke rodent model 24 h after ischemic reperfusion resulted in decreased levels of IgG, which is known to cross the compromised rodent BBB and leak into the brain parenchyma [70]. MMP activity, which is associated with dysfunction of tight junctions between endothelial cells of the BBB [70], was also decreased in rodents that received NSC transplants following stroke [70]. In another study, transplantation of adjudin-preconditioned mouse embryonic NSCs into the ipsilateral striatum at 24 h after MCAO in adult mice resulted in decreased infarct volume and amelioration of BBB disruption, which was marked by significantly decreased Evans Blue dye and IgG leakage into the brain parenchyma [204]. These results suggest that NSCs are able to preserve the integrity of the BBB and help prevent further damage to the CNS following stroke [70, 204]. Since tPA is known to exacerbate neural cell death and BBB damage beyond the 4.5 h window, using NSC transplantation to preserve the integrity of the BBB could have immense clinical applications for extending the narrow therapeutic window of tPA to benefit a greater population of stroke patients.

#### Inflammation

**Fig. 2:**
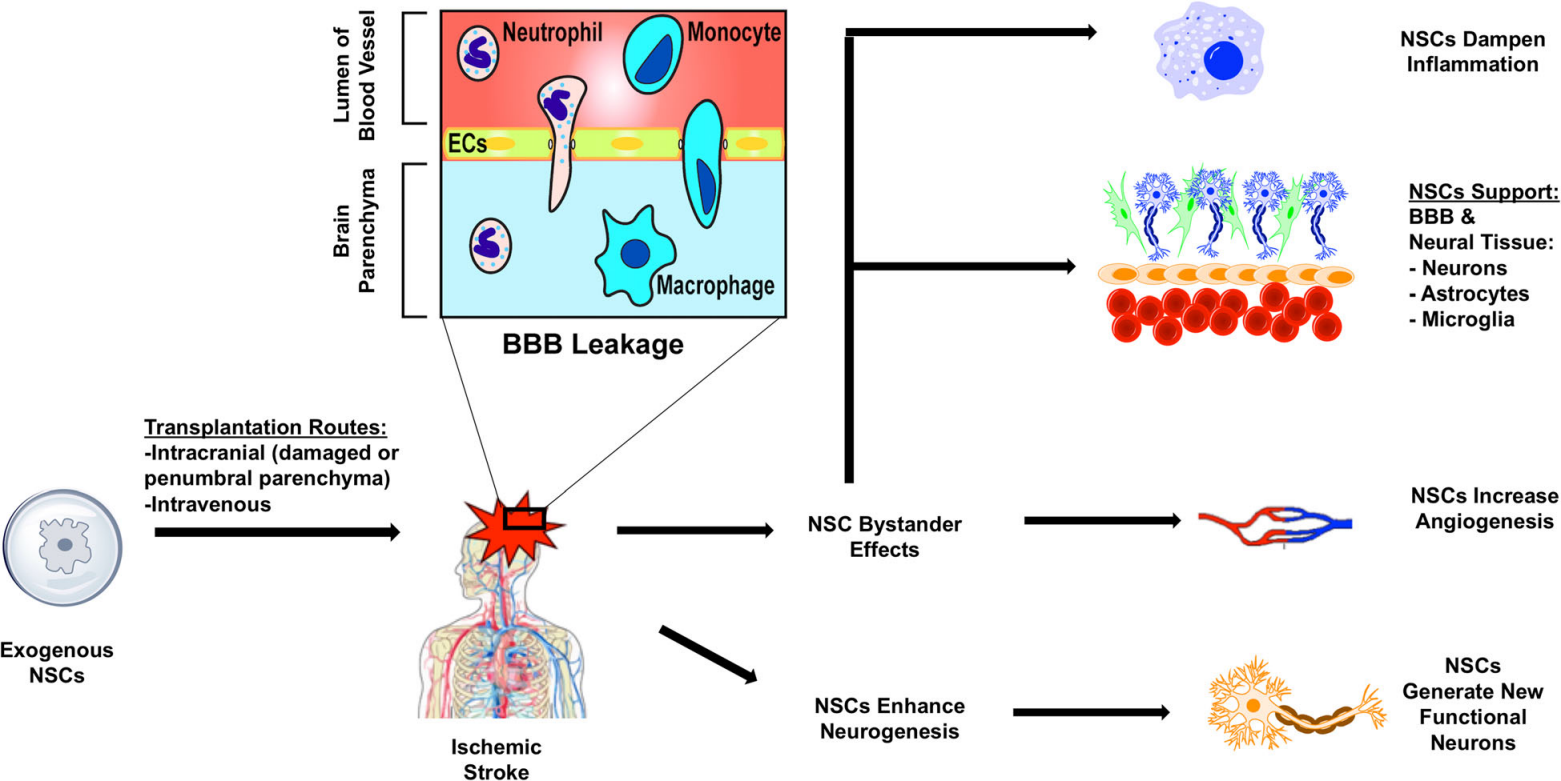
Schematic diagram of NSC transplantation illustrating multiple therapeutic benefits in ischemic stroke. Exogenous NSCs can be transplanted intravenously or directly into the damaged or penumbral parenchyma. NSCs can replace damaged neural cells in ischemic brain tissue. NSCs may also help to prevent further tissue damage in ischemic stroke through their bystander effects. These bystander effects include support of the blood-brain barrier (BBB), neurons, astrocytes, and microglia, as well as increased angiogenesis, and modulation of immune responses that result from ischemic insult. *Abbreviations*: *EC* endothelial cell, *NSC* neural stem cell

As mentioned above, ischemic stroke results in increased CNS inflammation as a result of activated astrocytes and microglia in addition to peripheral immune cell infiltration through the compromised BBB. Transplantation of NSCs in the subacute phase of ischemic stroke may also improve outcome by dampening this inflammatory cascade [177, 205, 206] (Fig. 2). Specifically, NSCs protect against further CNS damage by downregulating inflammatory regulator molecules such as TNF-α, monocyte chemoattractant protein-1 (MCP-1), IL-1β, IL-6, and Iba-1 [177, 206]. For example, hiPSC-NSC transplantation results in significantly lower amounts of Iba-1^+^ and ED1^+^ cells within and around the ischemic core in the stroke mouse model [207]. Animals in the transplanted group also had lower levels of glial fibrillary acidic protein-positive (GFAP^+^) astrocytes [207]. Similarly, NSC-treated mice had significantly reduced numbers of CD45^+^ and Iba-1^+^/MHC II immune cells within the brain post-stroke [206]. These results support the notion that NSCs can aid in functional recovery from ischemic insult by manipulating the extracellular microenvironment and decreasing inflammation.

### Long-term outcome of NSC transplantation at the early (subacute) stroke phase

The goal for transplantation of NSCs in the early subacute phases of stroke is neuroprotection from the cytotoxic microenvironment following ischemia. However, early NSC transplantation is also able to elicit neurorestorative mechanisms to improve long-term outcome in the days and weeks following initial ischemic insult.

#### Improved neurological outcome

NSC transplantation in the early stroke phase has also been demonstrated to improve behavior: an indication of functional recovery and the ultimate readout of any stroke therapy. Evaluation of neurological function using modified Neurologic Severity Score (mNSS) tests showed that when hNSCs were transplanted early into the rat brain (1 d after stroke), hNSC-transplanted rats showed significantly improved behavioral scores and performance 2 weeks after transplantation [208]. Also, studies showed that, by 48 h post-injury, neurological function rapidly improved in mice that received hNSC transplantation at 24 h after MCAO compared with non-transplanted stroke controls [69]. Transplanted mice showed improvement in the forepaw adhesive removal test (which assesses sensorimotor deficits), the beam walk test for assessing balance, and the rotarod test for evaluating balance and motor coordination. Recovery of behavioral function persisted through a month of monitoring [69], suggesting that early intervention is paramount for achieving lasting positive outcomes in stroke patients. In support of these results, other studies have demonstrated that engrafting iPSC-NSCs into mouse brains 24 h after MCAO improves long-term neurological outcome [70] and that transplantation of exogenous primary NSCs [69] or iPSC-derived NSCs [70] into the striatum and cortex of stroke animals results in improved behavioral tests and recovery [69, 70, 209]. Also, in a rodent stroke model, intracranial transplantation of NSCs directly into the hippocampus (where neurogenesis is ongoing) facilitated rapid migration to damaged areas and improved recovery after stroke [69, 70]. Another study showed that conditionally immortalized NSCs, murine NSC line clone 36 (MHP36), promoted functional recovery following intracranial transplantation 2 d after MCAO in mice [210]. When analyzed at various time points until 28 d post-stroke, engrafted animals from this study showed improved foot-fault function assessed by the ladder rung test and also improved asymmetry scores in the spontaneous forelimb (cylinder) test [210]. In other studies, transplantation of NSCs at later time points in the subacute phase of stroke (3–4 d after MCAO) also resulted in reduced infarct volume and improved neurological outcome compared with controls [211–213]. Taken together, these preclinical studies clearly demonstrate that NSC transplantation in the subacute phase of stroke significantly improves neurological and behavioral recovery.

#### Angiogenesis

NSC transplantation may help to facilitate recovery from stroke by promoting angiogenesis, which is coupled to neurogenesis [160]. In one study, early transplantation of hNSCs (1 d) following stroke enhanced angiogenesis in rats [208]. At day 14 after NSC transplantation, the number of BrdU^+^/vWF^+^ (von Willebrand factor-positive) proliferating endothelial cells was increased in the ischemic region of NSC-engrafted rats, suggesting increased angiogenesis [208]. Another study showed that early transplantation (6 h after MCAO) of mouse NSCs (preconditioned with IL-6) into the cortex promoted angiogenesis marked by increased density of lectin-perfused blood vessels at 14 d after transplantation [209]. In another study, intravenous administration of magnetically tagged, human fetal NSCs 24 h after MCAO also resulted in an increased number of cells positive for vWF and augmented angiogenesis when brains were analyzed at 28 d post-injury [214]. In aged rats, transplantation of mouse embryonic NSCs into the ipsilateral striatum 24 h after MCAO was able to reduce infarct volume, at least partially through increased VEGF expression and enhanced angiogenesis [215]. These studies clearly indicate that NSC therapy during the early stroke phase is able to have long-lasting beneficial effects on neovascularization.

#### Neurogenesis

In addition to angiogenesis, studies indicate that transplantation of NSCs in the early stroke phase may benefit long-term recovery through functional neuronal replacement, either directly or through augmentation of the endogenous neurogenic response [70, 214–222]. For instance, implantation of exogenous human fetal NSCs into the ipsilateral striatum 48 h after MCAO in rats resulted in subpopulations of engrafted cells that differentiated to neuroblasts and mature neurons at 6 and 14 weeks in addition to increased amounts of proliferating and migrating neuroblasts from the SVZ [216].

Other studies have reported modest neuronal replacement following NSC transplantation in the early phases of stroke as well. When hNSCs were injected on the same day (D0) following transient forebrain ischemia in rats, intravenously engrafted hNSCs differentiated into mature neurons to replace lost neural cells in the adult hippocampus of human–rat neural chimeras [219, 220]. Similar results have been observed with intravenously transplanted, magnetically targeted hNSCs at 24 h after MCAO in rats [214]. Transplanted hNSCs also give rise to astrocytes in the rodent brain following transient forebrain ischemia [214, 219, 220] and intracranial hemorrhage [221]. Transplantation of mouse NSCs into the ipsilateral striatum of rats 24 h after MCAO also augmented endogenous neurogenesis and reduced infarct volume [215, 222]. Of note, even though limited cell replacement has been observed in studies, a considerable amount of time is required for NSCs to differentiate into functional neurons, and there is still not enough evidence that cell replacement is vital for NSC-mediated recovery. For instance, a different study demonstrated that intracranial iPSC-NSC engraftment at 24 h post-stroke resulted in survival of engrafted cells at 30 d post-transplant, but the vast majority of donor cells remained as nestin-positive NSCs, and only a small percentage of the exogenous hiPSC-NSCs co-expressed the neuronal marker TuJ-1 [70]. Thus, these findings clearly suggest that neural replacement may not be a main contributor to beneficial stroke outcome after NSC transplantation in the subacute stroke phase.

### NSC transplantation at chronic stroke phase

Current clinical trials focus on the effect of stem cells on neurorestoration by administering cells during the chronic stroke stage when inflammatory signals are diminished. In rodent stroke models, pro-inflammatory cytokines were highly expressed in the acute/subacute stages of MCAO but decreased over time and returned to almost normal baseline levels 12 d after MCAO [223]. Stem cell transplantation at delayed time points (e.g., 7 d after stroke) in rodent models can avoid the cytotoxic environment in the acute/subacute phases of IR [224, 225]. Thus, transplantation of NSCs in this chronic phase of stroke may be an attractive model to test “neurorestorative” strategies to repair the damaged CNS. Indeed, preclinical research suggests that multiple actions of NSCs offer great promise in treating the chronic phase of stroke through multiple mechanisms, including enhancement of neurogenesis and angiogenesis, mediated at least in part by paracrine signaling through growth factors and neurotrophins.

#### Angiogenesis

In addition to enhancing the endogenous neurogenic response, several lines of evidence indicate that exogenous NSC transplantation during the chronic phase of stroke facilitates angiogenesis and neovascularization. For example, transplanting iPSC-NSCs into the penumbra intracranially 7 d after stroke in neonatal rat brains resulted in greater expression of VEGF in the peri-infarct region [226]. At 21 d post-stroke (14 d post-transplant), iPSC-NSC engraftment increased angiogenesis, as shown by the increased number of BrdU/collagen IV co-labeled cells in the vessel basement membrane in the peri-infarct area compared with stroke controls [226]. In support of these results, a study that modeled ischemic stroke in adult nude male rats with distal MCAO demonstrated that transplantation of NSCs at 7 d post-injury resulted in delayed “spatio-temporal” enhancement of neovascularization, which was dependent upon NSC expression of human VEGF [227]. Transplantation of IL-6 preconditioned fetal murine NSCs into the peri-infarct cortex 7 d after MCAO also enhances angiogenesis through STAT3-mediated upregulation of VEGF [209], and transplantation of SDF-1α–overexpressing iPSC-derived NSCs in the same brain region at 7 d post-focal ischemia also displayed significantly increased angiogenesis and recovery [228]. In another study using the microsphere-induced cerebral embolism rat model, animals that received intravenous injection of NSCs on day 7 after cerebral ischemia showed improved angiogenesis by day 28 compared with controls [229]. Moreover, animals that received exogenous NSCs displayed greater expression levels of VEGF, and its receptor VEGFR2 in brain capillaries [229]. Similar results have been observed with the CTX0E03 human immortalized NSC cell line [230], whereby stroke-damaged mouse brains that received CTX0E03 engraftments demonstrated significant increases in VWF^+^ microvessels compared with vehicle-treated brains 7 d after transplant [231]. Taken together, these studies suggest that exogenous NSCs largely facilitate recovery in the chronic phase of stroke through augmentation of angiogenesis and neovascularization, which is likely a result of increased VEGF paracrine signaling.

#### Neurogenesis

Disability from stroke is ultimately a result of mass cell death and tissue loss in the CNS. Therefore, direct replacement of lost brain cells through neurogenesis is an obvious therapeutic mechanism of NSC transplantation in the chronic phase of stroke. Several preclinical studies have demonstrated that transplanted NSCs differentiated into mature neurons and integrated with host neuronal circuitry in the chronic phase of stroke and engrafted animals showed improved functional and behavioral recovery [176, 226, 232–235]. For instance, engrafted iPSC-NSCs at 7 d after stroke in neonatal rat brains differentiated into neurons showing significantly greater numbers of BrdU/NeuN^+^ cells at 21 d post-stroke [226]. Rats that received the iPSC-NSC engraftments also performed better on the vibrissae-elicited forelimb placement test [226], which examines forepaw motor function and whisker somatosensation as proxies for neurological recovery following stroke [236, 237]. Another study demonstrated that hiPSC-derived neuroepithelial-like stem cells transplanted 9 d after MCAO were able to differentiate into mature neurons with electrophysiological activity in stroke brains of rats and mice [176]. Moreover, at 4 weeks post-transplantation, engrafted cells were receiving synaptic input from surrounding endogenous neurons, extended their axonal projections, and improved motor recovery in behavioral tests [176]. Transplantation of human ESC-NSCs 14 d after MCAO enhanced endogenous neurogenesis, measured by expression of doublecortin (Dcx), a marker for immature neurons, at 60 d in the ipsilateral SVZ in both young and aged rats [232]. Delayed transplantation of human ESC-derived neural precursor cells also reduced infarct volume and improved behavioral outcome in rats [233]. Transplantation of CTX0E03 in rats 4 weeks after MCAO significantly increases endogenous proliferation of Dcx^+^ neuroblasts in the striatum of the ischemic host brain [234] and leads to dose-related improved behavioral and functional improvements [235].

Of note, not all transplanted NSCs are able to survive and differentiate into mature neurons within the stroke-damaged brain [238]. Transplantation of NSCs after 7 d showed that grafted NSCs made synaptic connections with host neurons and displayed electrophysiological properties of mature neurons [176]. However, the number of NSCs that successfully differentiated and survived in the host brain was lower than expected [176]. Some studies have reported that NSC-engrafted brains from adult rats showed a significantly decreased number of donor cells from 4 to 12 weeks after stroke [177]. As mentioned above, differentiation of NSCs into functional CNS cell types within the stroke-damaged brain is a feat that requires significant time in a hostile microenvironment. Even though modest replacement of functional neurons has been observed in some NSC transplantation studies, it is still unclear whether cell replacement mechanisms are largely responsible for improved outcome. In fact, reports that low graft survival is still associated with improved recovery in the chronic stroke phase suggest that the therapeutic potential of NSC transplantation may place greater emphasis on augmentation of host neurogenesis and angiogenesis.

## Conclusions

Our ability to address neurovascular diseases will require multifaceted solutions, including pharmacological, cell replacement, genetic, and tissue engineering. The fundamental biological attributes of NSCs may likely be harnessed to circumvent some of the obstacles that currently exist in treating stroke. NSCs can be used adjunctively with other interventions such as tPA. These interventions may indeed work synergistically with each other. Specifically, NSCs show anti-inflammatory, anti-apoptotic, pro-angiogenic, and pro-regenerative effects, which can ameliorate the adverse side effects associated with tPA treatment in stroke patients. NSC transplantation in preclinical models also clearly improves functional recovery after stroke. However, more research is still needed to contribute to successful clinical trials and expand patient inclusion criteria for stroke treatment. For example, diabetes not only increases the risk of having a stroke but increases post-stroke mortality by three to four times [239, 240]. Owing to enhanced risk of BBB damage, patients with diabetes are also not able to receive tPA treatment [239]. In rodent models of stroke, diabetic mice have a more rapid increase in MMP-9 mRNA and protein activity post-stroke compared with controls [241]. Another mechanism by which diabetes increases risk of damage is through increased inflammation, as diabetic mouse models have shown increased inflammatory markers [241]. Therefore, more therapies are needed in order to treat stroke in patients with comorbidities.

Although much of the preclinical research on stem cell transplantation for stroke therapy has yielded promising results, certain safety concerns must also be properly addressed. For instance, one study that transplanted iPSCs into the ipsilateral striatum 24 h after MCAO in mice reported tumorigenesis from the transplanted cells, which was exacerbated in ischemia-damaged brains versus sham-operated brains [242]. Even though iPSC transplantation was able to supply Dcx^+^ neuroblasts and some mature neurons, recovery was still delayed in MCAO mice that received iPSCs compared with MCAO control mice that received only phosphate-buffered saline [242]. Nevertheless, these results highlight the importance of minimizing tumorigenic potential of stem cells for treatment of ischemic stroke.

Clinically, stem cell therapy is currently aiming for stroke rehabilitation by transplanting stem cells during the later recovery periods following stroke, and NSCs are beginning to gain attention for human trials [243–245]. For example, a phase 1 clinical trial with hNSCs showed no safety concerns and some promising signs of efficacy [244]. However, stem cell therapy for the subacute phase may benefit more stroke patients by ameliorating early-phase stroke injury and subsequently improving chronic-phase stroke outcome. Therefore, there is a major need for additional studies on early-phase post-stroke BBB disruption, how tPA contributes to it, and where to therapeutically target this pathological mechanism to increase the therapeutic window of tPA and ultimately improve stroke outcome.

## Abbreviations

BBB: Blood-brain barrier
BDNF: Brain-derived neurotrophic factor
CNS: Central nervous system
CXCR4: C-X-C chemokine type 4 receptor
EGF: Epidermal growth factor
ESC: Embryonic stem cell
FGF: Fibroblast growth factor
hiPSC: Human induced pluripotent stem cell
hNSC: Human neural stem cell
iPSC: Induced pluripotent stem cell
IR: Ischemia-reperfusion
MCAO: Middle cerebral artery occlusion
MMP-9: Matrix metalloproteinase-9
NSC: Neural stem cell
NTF: Neurotrophic factor
NVU: Neurovascular unit
SDF-1α: Stromal cell–derived factor 1-alpha
STAIR: Stroke Treatment Academic Industry Roundtable
SVZ: Subventricular zone
TGF-β: Transforming growth factor-beta
Th1: Type 1 T helper
Th2: Type 2 T helper
TNF-α: Tumor necrosis factor-alpha
tPA: Tissue plasminogen activator
VEGF: Vascular endothelial growth factor
